# Extracellular Vesicles Released by Bovine Alphaherpesvirus 1-Infected A549 Cells May Limit Subsequent Infections of the Progeny Virus

**DOI:** 10.3390/ijms26136181

**Published:** 2025-06-26

**Authors:** Yuanshan Luo, Hao Yang, Yike Huang, Renee V. Goreham, Xiuyan Ding, Liqian Zhu

**Affiliations:** 1 Key Laboratory of Microbial Diversity Research and Application of Hebei Province, College of Life Sciences, Hebei University, Baoding 071002, China; lys163001@163.com (Y.L.); yanghao11yyy@163.com (H.Y.); 19993880996@163.com (Y.H.); 2 School of Information and Physical Sciences, College of Engineering, Science and Environment, University of Newcastle, Callaghan, NSW 2308, Australia; renee.goreham@newcastle.edu.au

**Keywords:** BoAHV-1, extracellular vesicles, oncolytic virus, gD

## Abstract

Bovine alphaherpesvirus 1 (BoAHV-1) is a promising oncolytic virus that can infect the human lung carcinoma cell line A549. In an effort to adapt the virus to grow more rapidly in these cells through the serial passaging of viral progeny, we were unsuccessful. Here, we found that extracellular vesicles (EVs) secreted by BoAHV-1-infected A549 cells (referred to as EDVs) contain 59 viral proteins, including both viral structure proteins (such as gC and gD) and viral regulatory proteins (such as bICP4 and bICP22), as identified via a proteomic analysis. These EDVs can bind to and enter target cells, inhibit viral particles binding to cells, and stimulate the production of IFN-α and IFN-β in A549 cells. When EDVs are inoculated into rabbits via either the conjunctival sacs or intravenously, they can be readily detected in neurons within the trigeminal ganglia (TG), where they reduce viral replication and promote the transcription of IFN-γ. Furthermore, incorporation of the known anti-herpesvirus drug Acyclovir (ACY) into the EDVs leads to synergistically enhanced antiviral efficacy. Collectively, the EDVs exhibit antiviral effects by blocking viral binding to target cells and stimulating the innate immune response, thereby leading to the failure of the serial passaging of viral progeny in these cells, and these EDVs may serve as a promising vector for delivering drugs targeting TG tissues for antiviral purposes.

## 1. Introduction

Bovine alphaherpesvirus 1 (BoAHV-1) is member of the genus *Varicellovirus* in the subfamily *Alphaherpesvirinae* under the family *Herpesviridae*. It is an important viral pathogen, associated with a variety of inflammatory diseases in upper respiratory and genital tracts, causing substantial economic losses in the cattle industry [1,2,3]. Following infection of the mucosal epithelium in the oral, nasal, and ocular cavities, the virus invades the axons of sensory neurons and migrates to the trigeminal ganglia (TG) and brainstem, where it establishes a latent reservoir [4,5].

In 2010, the Mossman lab, for the first time, identified BoAHV-1 as a potential oncolytic virus, which is capable of infecting and killing various tumor cell lines, including human lung carcinoma A549 cells, while sparing healthy human cells [6]. The virus is non-infectious to humans, and its oncolytic efficacy does not depend on viral replication, as reported elsewhere [7,8]. These characteristics give it an advantage over other oncolytic virotherapies based on human viruses. As a result, it has attracted extensive studies focused on the development of BoAHV-1-based virotherapies. Using an A549 tumor xenograft mouse model, we previously demonstrated that the virus indeed exhibits antitumor effects. However, it has limited ability to spread within tumor tissues [9]. Even though it has been confirmed that its oncolytic effects do not depend on viral replication, we expected that if the virus could grow and spread more rapidly within tumor tissues, it would significantly enhance its oncolytic efficacy. In an effort to adapt the virus to grow faster and achieve higher yields in A549 cells, we attempted to perform the serial passaging of viral progeny in these cells. However, our attempts were unsuccessful, despite the fact that the virus grown in MDBK cells can productively infect A549 cells, leading us to question what happened to the progeny virus produced in A549 cells.

Extracellular vesicles (EVs) are lipid-derived nanovesicles with a size ranging from 40 to 160 nm, secreted by a variety of cells and widely found in many body fluids, including blood, urine, saliva, and semen. These vesicles play an essential role in intercellular communication because they contain nucleic acids, proteins, lipids, and other bioactive molecules that are crucial for both physiological and pathological processes within the body [10,11]. EVs have also been reported to play a role in the pathogenesis of numerous diseases, such as cancers [12], atherosclerosis and calcific aortic valve stenosis [13], and cardiovascular disease [14,15]. They have even been recognized as a “liquid biopsy” for neurological disorders [16]. To date, extensive studies have been conducted across a range of viruses to elucidate the roles of EVs in viral replication and infection-related disease progression. These studies have suggested that EVs released by virus-infected cells may exert various effects on viral replication, transmission, and immune evasion [11]. For example, EVs have been identified as key factors involved in the development of hepatocellular carcinoma induced by infections of hepatitis B virus (HBV) and hepatitis C virus (HCV) [17,18,19,20,21]. EVs promote the infection and pathogenicity of Japanese encephalitis virus [22]. EVs potentially regulates host immune responses and inflammation, thereby serving as pathology-related clinical indicators of COVID-19 infection [23]. Importantly, it has been reported that BoAHV-1 glycoprotein gB is incorporated into EVs, which in turn affect MHC-II molecules by retarding HLA-DR export to the plasma membrane [24]. This suggested that EVs are also involved in BoAHV-1 infection. In addition, EVs has been shown to be hijacked by HSV-1 to facilitate the establishment of latency [25,26], indicating that these EVs may inhibit HSV-1 productive infections. These findings prompted us to investigate whether EDVs released from BoAHV-1-infected A549 cells play a role in limiting the infection of the progeny virus.

Importantly, EVs have been extensively studied as drug delivery vectors. For example, the delivery of therapeutic VEGFA mRNA targeting hearts via EVs has been assessed in mice [27]. EVs secreted by umbilical cord blood-derived M1 macrophages can be used to deliver the known anti-cancer drug cisplatin, thereby enhancing its therapeutic efficacy in ovarian cancer [28]. Carboplatin-loaded EVs derived from drug-resistant (DR) clones of Y79 cells have been shown to significantly enhance carboplatin’s anti-tumor effects [29]. Interestingly, it has been reported that when antimicrobial drug rifampin-loaded EVs are modified with a brain-targeting peptide, their permeability to the blood–brain barrier is significantly improved [30].

In this study, EDVs derived from BoAHV-1-infected A549 cells were purified and characterized using various analyses, both in vitro and in vivo. Our data demonstrated that these EDVs exhibit anti-BoAHV-1 effects, as assessed both in cell cultures and a rabbit model. In terms of the mechanism, these EDVs have the capacity to bind to and enter target cells, thereby blocking viral binding to the cells. Additionally, they differentially regulate the production of interferons, both in vitro and in vivo, which may consequently restrict the ability of the progeny virus to replicate and spread during serial passages in these cells.

## 2. Results

### 2.1. Extracellular Vesicles (EDVs) Derived from BoAHV-1-Infected A549 Cells Contain a Variety of Viral Proteins

**Figure 1:**
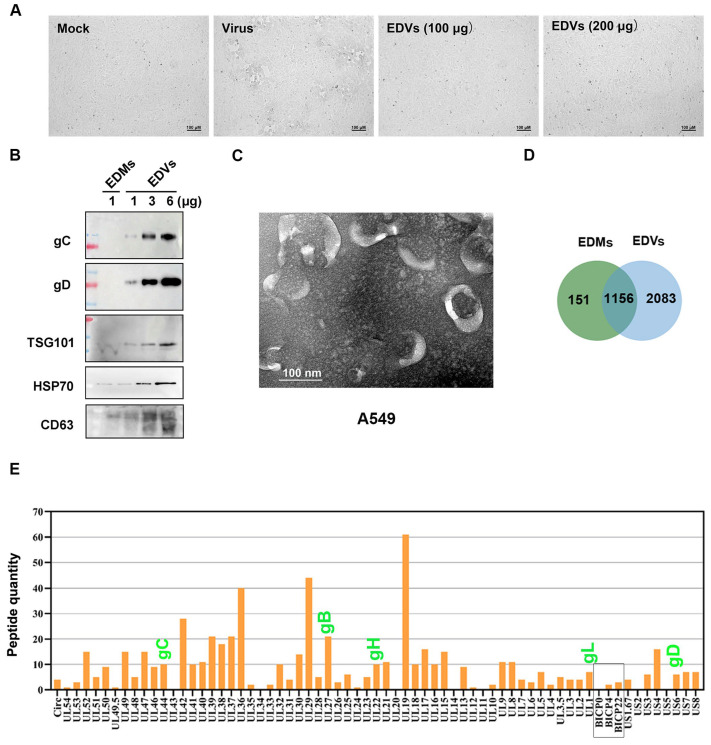
Identification of viral proteins in EDVs released from virus-infected A549 cells. (**A**) MDBK cells of confluent 24-well plates were either mock treated or treated with EDVs at the indicated doses for 24 h. As an infection positive control, the cells were infected with BoAHV-1 at an MOI of 1 for 24 h. Images shown are representative of three independent experiments. (**B**) Viral proteins gC and gD, along with host proteins TSG101 and CD63 (indicative markers of EVs), were detected in EVs derived from either mock-infected A549 cells (EDMs) or virus-infected A549 cells (EDVs) at increasing protein levels ranging from 1 to 6 μg. Detection was performed via Western blot using antibodies against gD (VMRD, cat# 1B8-F11, 1:2000), gC (VMRD, cat# F2, 1:2000), TSG101 (Abclonal, cat# A5789), and HSP70 (Abclonal, cat# A12948), respectively. All experiments were repeated three times. Data shown are representative of three independent experiments. (**C**) Representative images of purified EDVs observed under a Transmission Electron Microscope. The scale bar is 100 nm. (**D**) Total number of identified proteins in EDMs and EDVs using proteomics. (**E**) The identified viral proteins in EDVs using proteomics.

To determine whether EDVs released by virus-infected A549 cells contain viral proteins, EVs from either mock-infected (referred to as EDMs) or virus-infected (referred to as EDVs) cells were isolated using a commercial exosome isolation kit. Aiming to prevent the contamination of EDVs by viral particles, viral particles were excluded through ultracentrifugation prior to EDV purification. As shown in Figure 1A, the incubation of MDBK cells with EDVs resulted in the absence of the typical cytopathic effect (CPE) observed in virus-infected cells. This confirmed that infectious viral particles were successfully excluded as expected. Subsequently, the presence of viral proteins gD and gC in the isolated EDVs was detected via Western blotting using commercially available monoclonal antibodies. Numerous host proteins, such as CD9, CD63, CD81, Alix, TSG101, and HSP70, are typically incorporated into EVs and can serve as specific markers for these vesicles [31,32]. Here, we found that both TSG101 and HSP70, along with viral proteins gC and gD, were detected in the isolated fractions of EDVs (Figure 1B). The vesicle structures were clearly observed under a transmission electron microscope, as shown in Figure 1C, confirming that EVs were successfully purified using this commercial kit. To further identify the content of these EVs, both EDMs and EDVs containing 20 μg of protein were subjected to a proteomic analysis. A total of 1307 and 3239 proteins were detected in EDMs and EDVs, respectively (Figure 1D). Among these, 1156 proteins were commonly present in both EDVs and EDMs, while 2083 proteins were uniquely observed in EDVs. These findings suggested that virus infection altered the contents of EDVs. A total of 59 viral proteins were identified in EDVs (Figure 1E and Table 1), including gC, gD, gB, gH, and gL, which are virion-associated proteins known to be critical for virus entry [33]. Among these 59 proteins, UL19, the major capsid protein of BoAHV-1, was found to be the most abundant. Surprisingly, in addition to the viral structural proteins such as gD, gC, and gH, two immediate-early (IE) proteins, bICP22 and bICP4 (Figure 1E, highlighted with a frame, and Table 1), were also detected in EDVs. Of note, these IE proteins are viral regulatory proteins that are typically produced during virus replication to facilitate viral gene expression and are not usually incorporated into virions [34,35]. Nevertheless, they can also be released into the extracellular space through the cargo of EVs. The term “intensity” is commonly used to quantify the abundance of a protein or peptide within a sample in proteomic assays. When comparing the intensities derived from both EDVs and EDMs, we found that some peptides derived from UL42, UL37, UL27, UL5, and US4 were also observed in EDMs (Table 1). This suggests that these viral proteins may share some identical peptide sequences with certain host proteins.

Taken together, these findings indicate that BoAHV-1 productive infection in A549 cells significantly alters the content of released EDVs, with numerous viral proteins, including certain IE proteins, being incorporated into EDVs.

### 2.2. EDVs Can Be Internalized into Various Target Cells and Induce Production of Type I Interferons Such as INF-α and IFN-β

**Figure 2:**
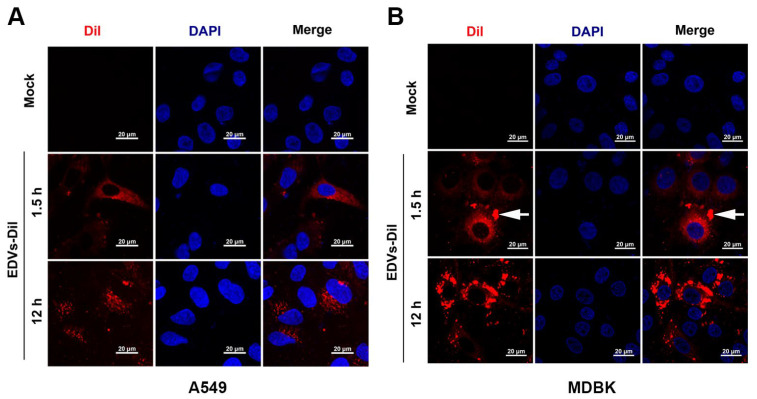
Characterization of whether EDVs can be internalized by BoAHV-1 permissive cells. A549 (**A**) and MDBK (**B**) cells were incubated with Dil-labeled EDVs for the indicated times at 37 °C. After three washes with PBS, the cells were fixed with 4% paraformaldehyde, and the nuclei were stained with DAPI. Then, the cell-associated or internalized EDVs were observed using a Leica confocal system (**A**) or a Zeiss confocal system (**B**) with a scale bar of 20 μm. The cells treated with PBS were used as a control. All experiments were repeated three times. These images shown are representative of three independent experiments. While arrows are indicative are dot-like structures of internalized Dil-labeled EDVs.

It is well established that the gD (US6) dimer, gH (UL22)-gL (UL1) heterodimer, and gB (UL27) trimer are essential components for herpesvirus entry. Mechanistically, gD binds to one of several entry receptors, such as Nectin-1, and triggers signal transduction to the gH–gL complex. This, in turn, activates the fusion protein gB, enabling it to insert its hydrophobic fusion loops into the cell membrane [36,37,38,39,40]. Given that gD, gH, gL, and gB were all present in EDVs (Figure 1E and Table 1), we investigated whether EDVs could bind to and enter host cells. To this end, both A549 and MDBK cells were incubated with fluorescent dye Dil (1,1′-dioctadecyl-3,3,3′,3′-tetramethylindocarbocyanine perchlorate)-labeled EDVs for 1.5 and 12 h, respectively, and then the fluorescence, indicative of EDVs, was examined via confocal microscopy. At 1.5 h post-incubation (hpi), Dil-labeled EDVs internalized by both A549 and MDBK cells were readily observed as dot-like vesicles within the cytoplasm (Figure 2A,B, middle panels, denoted by white arrow). After a 12 h incubation, prominent Dil puncta were clearly observed in the cytosol (Figure 2A,B, bottom panels), further confirming the internalization of these EDVs. Meanwhile, the fluorescence was rarely observed in mock-treated controls (Figure 2A, upper panels), which validates the specific staining. Collectively, these data demonstrate that EDVs can bind to and enter target cells of both A549 and MDBK.

**Figure 3:**
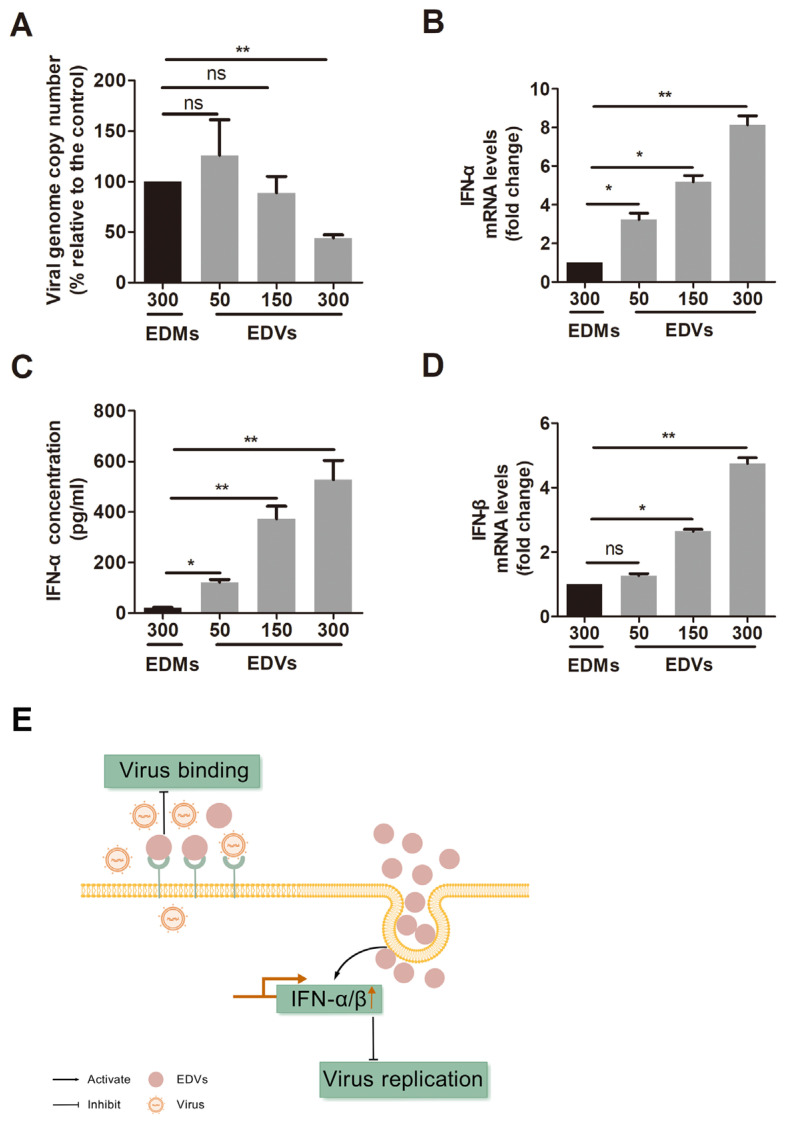
Characterization of the anti-BoAHV-1 effects of EDVs in cell cultures. (**A**) A549 cells in 6-well plates were pretreated either with either EDMs or EDVs at increasing concentrations ranging from 50 to 300 μg/mL for 1 h at 4 °C. Then, the cells were incubated with ice-cold virus at an MOI of 10 for 1 h at 4 °C. After three washes with ice-cold PBS, the cells were harvested, and total DNA was extracted using a host DNA purification kit. Then, the viral genome was detected via qPCR using primers targeting the viral gC gene. (**B**) A549 cells in 6-well plates were either mock treated with EDMs or treated with EDVs at the indicated concentrations for 24 h. The cells were collected, total RNA was purified, and the mRNA levels of INF-α (**B**) and INF-β (**D**) were detected via RT-qPCR. In parallel, the supernatants were collected and clarified via centrifugation. The protein concentrations of INF-α were detected using a commercial ELISA kit (Abclonal, cat#RK00092) (**C**). (**E**) Schematic of EDV-mediated antiviral effects. EDVs inhibit virus infection potentially by blocking the binding of virions to the cells and stimulating the expression of INFs, such as INF-α and INF-β. All experiments were repeated three times. Data shown are representative of three independent experiments. Statistical analyses were performed using Student’s *t*-tests (* *p* < 0.05, ** *p* < 0.01, ns not significant).

Furthermore, the pretreatment of A549 cells with ice-cold EDVs for 1 h efficiently blocked the binding of BoAHV-1 virions to A549 cells in a dose-dependent manner, as measured via quantitative PCR (Figure 3A). Specifically, it was reduced to approximately 44.3% following treatment by EDVs at a concentration of 300 µg/mL compared to the EDM-treated control. These findings suggest that EDVs can competitively inhibit the binding of viral particles to host cells.

EVs are potentially involved in the regulation of immune responses through various mechanisms [41]. We evaluated whether EDVs have the potential to stimulate the innate immune response by detecting IFN-α and INF-β. As shown in Figure 3B, treatment with EDVs led to a dose-dependent increase in IFN-α mRNA levels. Specifically, IFN-α mRNA levels increased to approximately 3.22-, 5.19-, and 8.13-fold with EDVs at concentrations of 50, 150, and 300 µg/mL, respectively. IFN-α protein levels in cell cultures treated with EDMs were 20.81 pg/mL, which increased to 120.83, 372.48, and 527.52 pg/mL following stimulation with EDVs at concentrations of 50, 150, and 300 µg/mL, respectively, as determined using an ELISA kit (Figure 3C). In addition, IFN-β mRNA levels increased to approximately 2.64- and 4.75-fold following treatment with EDVs at concentrations of 150 µg/mL and 300 µg/mL, respectively, as determined via RT-qPCR (Figure 3D). These results suggest that EDVs stimulate the expression of type I interferons, including IFN-α and IFN-β. Collectively, the EDVs may inhibit viral productive infection through two coupled mechanisms: preventing viral binding to host cells and subsequently activating the antiviral immune response, as evidenced by the enhanced expression of both IFN-α and IFN-β, as illustrated in Figure 3E.

### 2.3. EDVs Can Be Delivered into TG Neurons and Have the Capacity to Disrupt Virus Productive Infection In Vivo Partially via the Enhanced Induction of INF-γ

**Figure 4:**
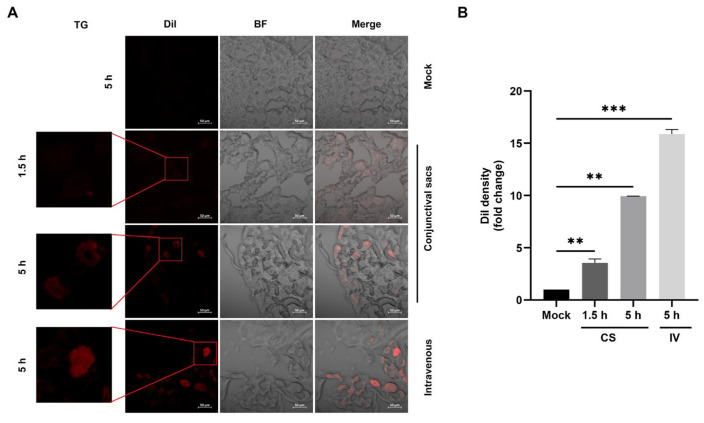
Detection of EDVs delivered into the TG neurons. (**A**) Dil-labeled EDVs at a dose of 400 μg was administered to rabbits via intranasal and ocular cavity routes or through both conjunctival sacs and intravenous routes. At 1.5 and 5 h after administration, the animals were euthanized, and tissues of the TG were quickly removed. Tissue sections approximately 10 μm in thickness were cut using a cryostat and directly mounted onto slides. Then, Dil fluorescence signals indicative of delivered EDVs were observed using a Zeiss confocal system. All experiments were repeated three times. The images shown are representative of three independent animals. (**B**) The fluorescence intensity of Dil was analyzed using free software Image J. The IntDen values per cell in EDV-treated TG neurons were normalized to that of the control, which was arbitrarily set as 1. Data shown are the means of three images. Significance was determined using a Student’s *t*-test (** *p* < 0.01, *** *p* < 0.001). CS: conjunctival sacs, IV: intravenous.

To test whether EDVs can be delivered into TG neurons in vivo, Dil-labeled EDVs were administered to rabbits via either the conjunctival sacs or intravenously. Then, frozen sections of TG tissues were observed under a confocal microscope. We found that Dil fluorescence puncta were readily detected in a subset of TG neurons as early as 1.5 h after the administration of EDVs via both routes (Figure 4, zoom in areas). At 5 h after administration, a subset of TG neurons was distinctly stained with Dil fluorescence, exhibiting prominent signals regardless of the administration routes (Figure 4, zoom in areas). A quantitative analysis revealed that the Dil fluorescence intensity increased to approximately 3.53-fold at 1.5 h and 9.94-fold at 5 h after administration through the conjunctival sacs, respectively. Additionally, it reached a 15.87-fold increase at 5 h following intravenous administration, compared to the mock-treated control group. This suggests that EDVs can be delivered into TG neurons via either conjunctival sacs or intravenously.

**Figure 5:**
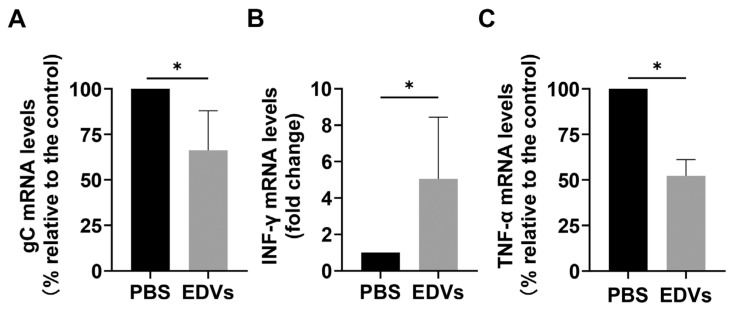
Evaluation of the antiviral effects of EDVs against BoAHV-1 lytic infection in vivo. Five-month-old female New Zealand White rabbits (*n* = 9) were lightly anesthetized with Xylazine followed by infection with 1 × 10^7^ PFU of BoAHV-1, as previously described [46]. At 4 hpi, approximately 600 μg of EDVs was administered to each rabbit, with a total of five, via both conjunctival sacs and intravenous routes. The remaining four rabbits received a mock treatment using PBS, serving as a treatment control. Each rabbit received four treatments with intervals of 8 h. Then, the TG was quickly removed for subsequent extraction of RNA. cDNA was transcribed, and mRNA levels of gC (**A**), IFN-γ (**B**), and TNF-α (**C**) were analyzed via relative quantitative real-time PCR, which was normalized to GAPDH. Normalized data are graphed as percentages of these virus-infected rabbits treated with EDVs relative to the mock-treated virus-infected control. Significance was determined using a Student’s *t*-test, and a *p*-value of less than 0.05 (* *p* < 0.05) was considered statistically significant.

Since our data indicated that EDVs can block virus entry in cell cultures (Figure 3A) and previous reports have shown that EVs released by HSV-1-infected cells activate innate immunity in recipient cells, leading to the suppression of virus replication in vitro [42], we were interested in characterizing whether EDVs have effects on virus lytic infection in vivo using a rabbit model. To this end, virus-infected rabbits were consecutively treated with either PBS control or EDVs for a total of four times, with intervals of 8 h between treatments. As a result, we found that viral lytic infection was inhibited by EDVs, as indicated by the reduced expression of the viral gene gC. Specifically, the gC mRNA levels decreased to 66.43% in EDV-treated rabbits compared to those in mock-treated controls (Figure 5A).

It has been reported that BoAHV-1 infection in calves stimulates the production of IFN-γ, a cytokine that activates the development of a cell-mediated immune response [43]. Additionally, IFN-γ has been shown to play an important role in protection against BoAHV-1 infection in vivo [44,45]. In the present study, we found that treatment with EDVs significantly increased the mRNA levels of IFN-γ in a rabbit model of viral infection. Relative to the mock-treated control, the IFN-γ mRNA levels increased to approximately 5.05-fold (Figure 5B). This enhanced expression of IFN-γ correlates with the observed antiviral effects of EDVs in the rabbit model.

When assessing the production of the canonical proinflammatory cytokine TNF-α using RT-qPCR, we found that TNF-α mRNA levels were significantly decreased following treatment with EDVs. Compared to mock-treated virus-infected rabbits, TNF-α mRNA levels were reduced to 52% in EDV-treated animals (Figure 5C). These finding suggested that EDVs can inhibit expression of the inflammatory-response-related cytokine TNF-α.

### 2.4. EDVs Enhance the Antiviral Effects of Acyclovir In Vitro

**Figure 6:**
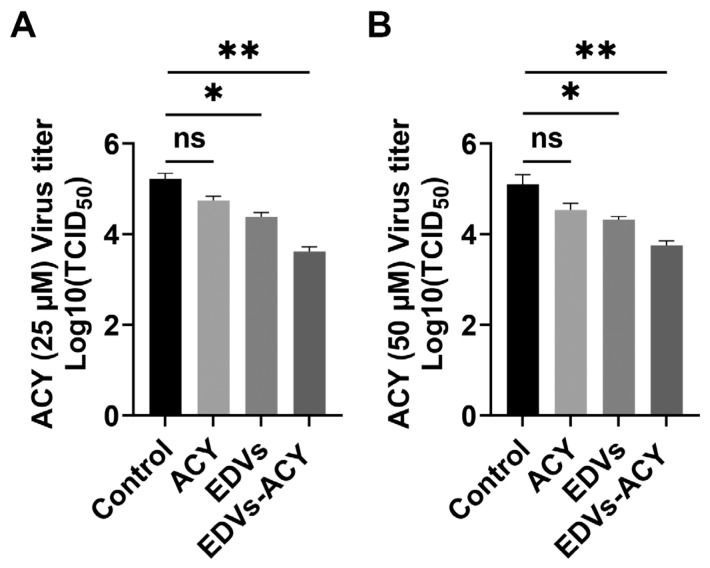
Characterization of the potential of EDVs as drug-delivery vectors. (**A**) MDBK cells in 6-well plates were treated with DMSO, 25 μM ACY, EDVs (200 μg/mL), or ACY (25 μM) incorporated into EDVs (ACY-EDVs) (200 μg/mL) for 1 h. Subsequently, the cells were infected with BoAHV-1 at an MOI of 1 for 1 h in the presence of DMSO, ACY, or ACY-EDVs at the concentrations described above. The cells were then washed three times with PBS, and fresh medium containing the indicated compounds was added at the same concentrations. After further incubation for 24 h, the cell cultures were harvested to determine virus titers, which were expressed as TCID_50_/mL. (**B**) MDBK cells in 6-well plates infected with the virus were treated with 50 μM ACY, EDVs (200 μg/mL), or ACY (50 μM) incorporated into EDVs (ACY-EDVs) (200 μg/mL) in the same manner as described in (**A**). After 24 h of infection, viral titers were determined using MDBK cells. All experiments were repeated three times. The data shown are the means of three independent experiments with error bars indicating standard deviations. Significance was assessed with a Student’s *t*-test (ns, not significant; * *p* < 0.05; ** *p* < 0.01).

EVs are increasingly recognized as therapeutic drug-delivery vehicles due to their amphipathic structure [47,48]. Given that EDVs can bind to and enter target cells, we investigated whether they could serve as a potential delivery vector to enhance the antiviral effects of the known antiviral drug, Acyclovir (ACY), when incorporated into EDVs using a sonication method, as described elsewhere [49]. In our experiments, ACY at a concentration of 25 μM exhibited minimal effects on virus replication. EDVs alone reduced the virus titer by approximately 0.84 log. However, when 25 μM ACY was incorporated into EDVs, the combination significantly reduced the virus titer by approximately 1.12 log and 1.60 log compared to 25 μM ACY alone and the mock-treated control, respectively (Figure 6A). The titers for the results expressed as log10 (TCID_50_/mL) shown in Figure 6A were as follows: 5.22 for DMSO control, 4.74 for 25 μM ACY, 4.38 for 200 μg/mL EDVs, and 3.62 for 200 μg/mL ACY-EDVs. When 50 μM ACY was assessed, it also demonstrated minimal effects on virus replication. Meanwhile, the virus titer was reduced by approximately 0.8 log and 1.34 log by EDVs-ACY (50 μM) compared to ACY alone and the mock-treated control, respectively (Figure 6B). The titers for the results expressed as log10 (TCID_50_/mL) shown in Figure 6B were as follows: 5.09 for DMSO control, 4.53 for 50 μM ACY, 4.32 for 200 μg/mL EDVs, and 3.75 for 200 μg/mL ACY-EDVs These results indicate that EDVs enhance the antiviral potency of ACY, although it does not show a dose-dependent mechanism. These results confirmed that EDVs have the potential to be developed as a drug-delivery vehicle, at least for generating anti-BoAHV-1 drugs, as the vector itself also possesses antiviral effects.

## 3. Discussion

Accumulating studies have shown that EVs may have a significant influence on viral binding to target cells across various viruses. For instance, EVs enriched in CD4^+^ molecules, released by T cells, can compete with HIV-1 for binding to host cells, thereby blocking viral entry into target cells [50]. EVs enriched in ACE2 proteins can interfere with infection by a broad range of SARS-CoV-2 strains [51]. Similarly, we found that EDVs derived from BoAHV-1-infected A549 cells can block viral binding to target cells (Figure 3A). This finding is directly supported by the observation that EDVs can bind to and enter target cells (Figure 2A,B), which may further indirectly correlate with our results that EDVs contain 59 viral proteins, including gD, gH, gL, and gB (Figure 1E, highlighted in green, and Table 1), which are known to mediate viral binding to receptors such as Nectin-1 [36,37,38,39,40,52]. It is worth noting that viral particles were excluded by ultracentrifugation prior to the isolation of EDVs. Consequently, the incubation of MDBK cells with these EDVs resulted in the absence of a typical CPE associated with viral infection, confirming that the isolated EDVs are free of infectious viral particles (Figure 1A). Thus, blocking viral binding to target cells exerted by the EDVs may further validate that the isolated EDVs may be free of infectious viral particles. Thus, the released EDVs are potential factors that contribute to restricting progeny virus infection by blocking virus binding to the host receptors.

It has been reported that human herpesvirus 6 (HHV-6) virions are released alongside intraluminal vesicles through the EV-release pathways [53]. Similarly, Epstein–Barr virus (EBV) and Kaposi’s sarcoma-associated herpesvirus (KSHV) have been shown to modify EVs to enhance their oncogenic potential [54,55]. Recently, it has been reported that CD63^+^ EVs induced during HSV-1 productive infection exhibit antiviral effects [25,56]. Here, we found that CD63 protein were was detectable in the isolated EDVs using Western blot assays (Figure 1B), and these EDVs also demonstrated antiviral effects (Figure 3A and Figure 6). However, the abundancy of CD63^+^ EVs in the EDVs remains to be determined, in the future. Interestingly, it has been reported that EVs released by HSV-1-infected cells contain the DNA sensor STING and exhibit antiviral effects, as assessed in cell cultures [25]. Here, we found that EDVs stimulate the transcription of IFN-γ in the context of BoAHV-1 lytic infection in a rabbit model (Figure 5B) and enhance the expression and release of INF-α and INF-β in cell cultures (Figure 3B,C). Thus, the stimulation of antiviral innate immune responses may represent a common capacity of EVs released from alpha herpesviruses, including HSV-1 and BoAHV-1. This character confers a significant advantage of EDVs to be used as an antiviral drug-delivery vehicle, which has been confirmed by the delivered antiviral drug ACY (Figure 6). In addition, the induction of interferon production may contribute to restricting progeny virus infection in A549 cells, which consequently leads to the failure of the serial passaging of viral progeny in these cells.

In fact, EVs from MDBK cells were also purified and identified. However, we found that, compared to EDVs, only minimal levels of the viral protein gD were detected from these EVs derived from MDBK cells. According to our preliminary studies, the abundance of EVs secreted by MDBK cells is much lower than that of A549 cells. Thus, these EVs were not subjected to extensive identification in the present study. However, a comparative study of the different compositions between these two types of EVs is an interesting topic that deserves an independent study in the future. Of note, in addition to the viral structure proteins such as gD, gC, and gH, some viral regulatory proteins, including bICP4 and bICP22, were also detected in the EDVs (Figure 1D, highlighted with frame, and Table 1). These regulatory proteins are typically intracellularly expressed during virus replication, which plays important roles in the regulation of viral gene expression, and are not incorporated into the viral particles. Here, for the first time, we reported that some viral IE proteins, such as bICP22 and bICP4, are secreted out of cells via the incorporation into EVs.

To date, significant efforts have been devoted to developing strategies for generating EVs as drug-delivery vectors targeting specific tissues. For instance, immature dendritic cells (DCs) that display a rabies virus glycoprotein (RVG)-derived peptide through plasmid transfection have been employed to endow the released EVs with brain-targeting capabilities [48]. This is because the viral peptide can specifically interact with corresponding receptors that are distributed in neuronal tissues. Thus, the interaction between viral proteins and host receptors may strictly determine the tissue tropism of the modified EVs. Following the same rationale, we have shown that EDVs released by BoAHV-1-infected A549 cells can mimic viral particles and exhibit tropism for neurons in the TG (Figure 4). This is possibly due to the enrichment of viral proteins in EDVs (Figure 1E and Table 1), which are able to mimic viral entry. Our findings suggest that the viral infection of certain cells may offer an alternative way to confer tissue tropism to EDVs.

BoAHV-1 is a potential oncolytic virus with great promise for the development of anti-tumor therapies. It has been confirmed that its oncolytic effects do not rely on viral replication [7,8]. Based on this rationale, the antiviral activity of EDVs released in virus-infected tumor cells observed in this study would not undermine its oncolytic effects. However, the ability to induce the production of interferons (IFNs) such as IFN-α, IFN-β, and IFN-γ would further enhance the virus’s oncolytic effects, as the triggered innate immune response plays a crucial role in anti-tumor activity. Thus, the EDVs may represent a promising source for the development of anti-tumor agents, warranting extensive studies in the future.

In summary, our findings demonstrate that EDVs demonstrate antiviral effects both in vitro and in vivo. In terms of the mechanism, the EDVs may inhibit viral productive infection through two coupled mechanisms: preventing viral binding to host cells and subsequently activating the antiviral immune response, as evidenced by the enhanced expression of both IFN-α and IFN-β in vitro, as well as IFN-γ in vivo. These antiviral properties may also account for the inability to successfully passage viral progeny in these cells. Additionally, we discovered that these EDVs hold significant potential as drug-delivery vehicles for the development of antiviral therapies targeting BoAHV-1.

## 4. Materials and Methods

### 4.1. Cells and Virus

A549 and MDBK cells were obtained from the Chinese Model Culture Preservation Center in Shanghai, China. They were cultured in Dulbecco’s Modified Eagle Medium (DMEMGibco, Thermo Fisher Scientific, Waltham, MA, USA) supplemented with 10% fetal bovine serum (FBS, Pricella, Wuhan, China) and routinely passaged. The BoAHV-1 strain NJ-16-1, isolated from commercial semen samples [57], was propagated in MDBK cells. Aliquots of the virus stock were stored at −80 °C until use.

### 4.2. Antibodies and Chemicals

The following antibodies were used in this study: TSG101 (cat# A5789) and HSP70 Rabbit pAb (cat#A12948) were ordered from Abclonal Technology (Woburn, MA, USA). BoAHV-1 gC mAb (cat#F2) and BoAHV-1 gD mAb (cat#1B8-F11) were supplied by VMRD Inc. (Pullman, WA, USA). HRP-conjugated goat anti-mouse IgG (cat# BF03001) and HRP-labeled goat anti-rabbit IgG (cat# BF03008) were procured from Biodragon (Suzhou, China).

### 4.3. Isolation of EVs

EVs were isolated from cell culture supernatants using a commercial exosome isolation kit (Invitrogen, Carlsbad, CA, USA, cat# 4478359), following the manufacturer’s protocol with minor modifications. Briefly, both cells and cell debris were removed from the collected supernatants of cell cultures through centrifugation at 4500× *g* for 5 min at 4 °C. The clarified supernatants were then subjected to a second round of centrifugation at 10,000× *g* for 10 min at 4 °C. At this stage, the clarified supernatants ideally contained both EVs and viral particles.

To isolate and remove viral particles from the supernatants, ultracentrifugation was performed following a modified protocol described elsewhere [58]. Specifically, the clarified supernatants as described above were subjected to ultracentrifugation at 20,000 rpm (68,600× *g*) using a Beckman SW41 Ti rotor for 1 h at 4 °C. The pellets attached to the ultracentrifugation tubes contained viral particles, while the supernatant was ideally free of viral particles.

Next, the virus-free supernatant was incubated with 0.5 volumes of the total exosome isolation reagent overnight at 4 °C, with gentle rotation on a roller shaker. This mixture was then centrifuged at 10,000× *g* for 1 h at 4 °C. The resulting pellets, which contained the EVs, were resuspended in 0.5 mL of 1× PBS. Aliquots of the EVs were stored at −80 °C until further use. The absence of viral contamination in the EVs was validated by incubating MDBK cells with the isolated EVs, which resulted in the absence of the typical cytopathic effect (CPE) induced by viral infection.

### 4.4. Western Blot Analysis

Protein lysates were prepared using the lysis buffer (containing 1% Triton X-100, 50 mM sodium chloride, 1 mM EDTA, 1 mM EGTA, 20 mM sodium fluoride, 20 mM sodium pyrophosphate, 1 mM phenylmethylsulfonyl fluoride, 0.5 g/mL leupeptin, 1 mM benzamidine, and 1 mM sodium orthovanadate in 20 mM Tris-HCl, pH 8.0), mixed with Laemmli sample buffer, and boiled for 10 min. The proteins were separated via 8% or 10% SDS-PAGE and then transferred onto PVDF membranes (Bio-Rad, Hercules, CA, USA, cat# 1620177). After blocking with 5% nonfat milk in PBS (pH 6.8) for 1 h at room temperature, the membranes were incubated with the indicated primary antibodies diluted in 5% bovine serum albumin in PBS (pH 6.8), overnight at 4 °C. Following extensive washing with PBS (pH 6.8), the membranes were incubated with appropriate secondary antibodies for 1 h at room temperature. After further washing with PBS, the protein bands were developed using Western ECL Substrate from NCM Biotech (Suzhou, China, cat# P10300).

### 4.5. Detection of EVs via Transmission Electron Microscopy (TEM)

Protein concentrations of EDVs were measured using the BCA assay kit (Beyotime Biotechnology, Shanghai, China, cat# P0010S) following the manufacturer’s protocol. The EDV solution was diluted to a concentration of 2 μg/mL with PBS and then loaded onto cropper grids and fixed with 2.5% glutaraldehyde. The samples were negatively stained with 2% uranyl acetate for 2 min. After drying under a lamp for 15 min, the samples were processed for observation via TEM.

### 4.6. Detection of EDV Internalization by Target Cells

MDBK and A549 cells were seeded into the 24-well plates containing coverslips and cultured in DMEM supplemented with 10% FBS until they reached 90% confluence. The EDVs, labeled with Dil (Beyotime Biotechnology, cat# C1991S) according to the manufacturer’s protocol, were then incubated with these cell cultures for 1.5 and 12 h, respectively. After three washes with ice-cold PBS, the cells were fixed with 4% paraformaldehyde for 10 min, and the nuclei were stained with 4′,6-diamidino-2-phenylindole (DAPI). Then, the coverslips with attached cells were removed from the 24-well plates for subsequent observation and imaging using a confocal microscope (Zeiss, Oberkochen, Germany or Leica, Wetzlar, Germany).

### 4.7. RNA Isolation and Quantification of mRNA via RT-qPCR

Either cells or TG rapidly removed following euthanasia were subjected to the purification of RNA using TRIzol LS reagent (Ambion, Waltham, MA, USA, cat# 10296010), according to the manufacturer’s instructions. A total of 1 μg RNA was used as a template for synthesizing first-strand cDNA with commercial random hexamer primers using the Thermoscript RT-qPCR system Kit (Invitrogen, cat# 11146-024) following the manufacturer’s protocol. The cDNA products were then used as templates for real-time PCR to measure the transcripts of viral gC, TNF-α, IFN-α, IFN-β, and INF-γ with the following primers: gC (forward reverse primer 5′-ACTATATTTTCCCTTCGCCCG-3′ and reverse primer 5′-TGTGACTTGGTGCCCATG-3′), TNF-α (forward reverse primer 5′-GACTTGGAGGGAGGGGATCT-3′ and reverse primer 5′-CATCTAGCCGTGGGGTTCTC-3′), IFN-α (forward reverse primer 5′-TCGCCCTTTGCTTTACTGAT-3′ and reverse primer 5′-GGGTCTCAGGGAGATCACAG-3′), IFN-β (forward reverse primer 5′-ACGCCGCATTGACCATCTAT-3′ and reverse primer 5′-AGCCAGGAGGTTCTCAACAAT-3′), INF-γ (forward reverse primer 5′-TTCTTCAGCCTCACTCTCTCC-3′ and reverse primer 5′-TGTTGTCACTCTCCTCTTTCC-3′), and glyceraldehyde-3-phosphate dehydrogenase (GAPDH) (forward reverse primer 5′-GTGGACCTGACCTGCCGCCT-3′ and reverse primer 5′-AGAGGAGTGGGTGGCACTGTGT-3′). An analysis of GAPDH mRNA served as an internal control. The expression levels of the tested genes were normalized to those of the GAPDH gene. Real-time PCR was performed using the LightCycler 96 fast real-time system (Roche, Tucson, AZ, USA, CHE). The relative mRNA level of each gene was calculated using the 2^−ΔΔCT^ method based on a comparison to the control.

### 4.8. Detection of Released IFN-α with ELISA

A549 cells in 6-well plates were treated with either EDMs or EDVs for 24 h. The supernatants were then collected and briefly clarified via centrifugation at 12,000 rpm for 5 min. IFN-alpha protein levels in the supernatants were detected using the IFN-alpha ELISA Kit (ABclonal, Woburn, MA, USA, cat#: RK00092).

### 4.9. Animal Experiment

Administration of EDVs to rabbits: EDVs were administered to rabbits to investigate their potential as a drug-delivery vehicle. Female New Zealand White rabbits, approximately 2 months old, were lightly anesthetized with Xylazine (MedChemExpress, Monmouth Junction, NJ, USA) (1.5 mg/kg, intramuscularly) and then administered 400 μg of Dil-labeled EDVs via either the conjunctival sacs or intravenously. At 1.5 and 5 h post-administration, the rabbits were sacrificed following euthanasia. TG were rapidly removed following euthanasia for a subsequent analysis of EDV-Dil localization within neurons, which was analyzed using a Zeiss confocal system.

Treatment of virus-infected rabbits using EDVs: three-month-old female New Zealand White rabbits were lightly anesthetized with Xylazine (MedChemExpress, Monmouth Junction, NJ, USA) (1.5 mg/kg, intramuscularly) and infected with 1 × 10^7^ PFU of BoAHV-1 virus through both conjunctival sacs and intravenous routes, as previously described elsewhere [46]. At 4 hpi, approximately 600 μg of EDVs was administered to each rabbit via the same routes as infection. The rabbits received a total of four EDV treatments, with an 8 h interval between each treatment. At 36 hpi, the rabbits were sacrificed following euthanasia. Then, the TGs were rapidly removed for subsequent analysis using RT-qPCR. As a control, the infected rabbits were treated with PBS using the same treatment manner as described above.

Animal care and study procedures adhered to the guidelines of the Animal Research Ethics Board of Hebei University (Approval number 2022-009103 and 2022-009104) and were conducted in accordance with the Guide for the Care and Use of Laboratory Animals by the National Research Council. Rabbits were provided by Tonghui Experimental Animals (Baoding, China).

### 4.10. Detection of Internalized Dil-Labeled EDVs in Neurons from the TG

The freshly removed TG samples were fixed in 4% paraformaldehyde at 4 °C for 24 h. Subsequently, the tissues were dehydrated in a solution of 30% sucrose (prepared in PBS) for an additional 24 h. The tissues were then embedded in optimal cutting temperature compound and stored at −20 °C. Tissue sections, approximately 10 μm in thickness, were cut using a cryostat and directly mounted onto slides. The Dil fluorescence signals, indicative of delivered EDVs, were observed using a Zeiss confocal system. The Dil fluorescence intensity was quantitatively analyzed using free software Image J as described elsewhere [59].

### 4.11. Statistical Analysis

All data presented in this study are expressed as the mean ± standard deviation (SD). Statistical analyses were performed using GraphPad Prism (version 8). Significance was determined using a Student’s *t*-test. A *p*-value less than 0.05 (∗ *p* < 0.05) was considered statistically significant.

## Figures and Tables

**Table 1:** The identified viral proteins in EDVs using proteomics.

Gene Name	Intensity EDMs	Intensity EDVs	Protein Description
Circ		1471470	circ protein
UL54		1782460	UL54 protein GN = UL54
UL53		338753	glycoprotein K GN = UL53
UL52		1431790	component of DNA helicase/primase complex GN = UL52
UL51		919147	virion protein GN = UL51
UL50		15733500	deoxyuridine triphosphatase GN = UL50
UL49.5		3110740	virion protein (membrane) GN = UL49.5
UL49		31395000	virion protein (tegument) GN = UL49
UL48		12708800	alpha-TIF protein GN = UL48
UL47		11379300	virion protein (tegument GN = UL47
UL46		6599390	virion protein (tegument) GN = UL46
UL44		10158900	glycoprotein C GN = UL44
UL42	43852900	261308000	processivity factor for DNA polymerase GN = UL42
UL41		4411390	virion host shutoff factor (tegument) GN = UL41
UL40		15975900	ribonucleotide reductase small subunit GN = UL40
UL39		8403990	ribonucleotide reductase large subunit GN = UL39
UL38		41214900	capsid protein GN = UL38
UL37	811350	2325140	virion protein (tegument) GN = UL37
UL36		4044840	very large virion protein (tegument) GN = UL36
UL35		7290220	capsid protein GN = UL35
UL33		133690	UL33 protein GN = UL33
UL32		3502420	UL32 protein GN = UL32
UL31		3071340	UL31 protein GN = UL31
UL30		1724140	DNA polymerase GN = UL30
UL29		116502000	major DNA binding protein GN = UL29
UL28		1123390	ICP18.5 assembly protein GN = UL28
UL27	489268	2577140	glycoprotein B GN = UL27
UL26		1916690	serine protease (capsid) GN = UL26
UL25		537027	UL25 protein GN = UL25
UL24		325064	virion protein GN = UL24
UL23		1908770	thymidine kinase GN = UL23
UL22		1593410	glycoprotein H GN = UL22
UL21		12896500	virion protein GN = UL21
UL19		63436600	major capsid protein GN = UL19
UL18		32282100	capsid protein GN = UL18
UL15		3017310	UL15 protein GN = UL15
UL17		5378610	UL17 protein GN = UL17
UL16		12515300	virion protein GN = UL16
UL13		1919140	virion serine/threonine protein kinase GN = UL13
UL12		1651800	deoxyribonuclease GN = UL12
UL10		122819	glycoprotein M GN = UL10
UL9		6760560	replication origin binding protein GN = UL9
UL8		2961340	component of DNA helicase/primase complex GN = UL8
UL7		1563210	UL7 protein GN = UL7
UL6		497820	UL6 protein GN = UL6
UL5	2552160	1823880	component of DNA helicase/primase complex GN = UL5
UL4		2357470	virion protein GN = UL4
UL3.5		6871300	UL3.5 protein GN = UL3.5
UL3		2245320	phosphoprotein GN = UL3
UL2		1734430	uracil DNA glycosylase GN = UL2
UL1		1779770	glycoprotein L GN = UL1
BICP4		302473	immediate-early transactivator protein (cell nucleus) GN = BICP4
BICP22		1552040	immediate-early and late transrepressor protein (cell nucleus) GN = BICP22
US1.67		4648000	US1.67 protein GN = US1.67
US3		1863100	virion serine/threonine protein kinase GN = US3
US4	761729	45762600	glycoprotein G GN = US4
US6		3996870	glycoprotein D GN = US6
US7		4721730	glycoprotein I GN = US7
US8		2426910	glycoprotein E GN = US8

## Data Availability

The authors declare that all the data are available upon request.
